# Impact of prematurity on lifelong cardiovascular health: structural and functional considerations

**DOI:** 10.1038/s44325-024-00002-0

**Published:** 2024-04-05

**Authors:** Ryan P. Sixtus, Rebecca M. Dyson, Clint L. Gray

**Affiliations:** 1 Cardiff School of Biosciences, Cardiff, CF10 3US UK; 2 School of Biomedical Sciences, Newcastle, NSW Australia; 3 Gilles McIndoe Research Institute, Wellington, New Zealand; 4 Department of Paediatrics and Child Health, University of Otago, Wellington, New Zealand

**Keywords:** Cardiovascular diseases, Hypertension

## Abstract

The aetiology of preterm cardiovascular disease formation appears different from that of traditional population. Within the ‘traditional’ population cardiovascular disease formation is driven by functional stressors (e.g., diet, smoking). Whereas preterm cardiovascular disease risk is driven by structural changes incurred at birth. Much of the proliferative growth in the developing heart and major vessels ceases at birth, leading to permanently reduced dimensions compared to their term-born cohort. These structural changes take a back seat to functional and clinical complications within the neonatal period, but become increasingly pronounced from adolescence, at which point functional decompensation can be observed. While the cause may differ from ‘traditional’ populations, the eventual disease outcomes do not, leading them to be an overlooked population. This means that aetiology, and thus, treatment options may be very different due to the underlying mechanisms. Here, we propose that the structural cause of preterm-associated cardiovascular disease is apparent and observable early in life. Understanding the differences in cardiovascular disease aetiology may therefore aid in the early treatment of preterm-associated cardiovascular disease risk.

## Introduction

Cardiovascular diseases (CVD) are the leading cause of mortality, claiming an estimated 19.1 million lives annually^1^. CVDs comprise heart failure, ischaemic heart disease, coronary artery disease, cerebrovascular disease, and various illnesses of the heart and blood vessels^2^. More than four out of five CVD-related deaths are caused by heart attacks and strokes, and one-third of these untimely deaths occur in those under the age of 70^3,4^. The most significant behavioural risk factors for cardiovascular disease and stroke include a poor diet, physical inactivity, cigarette use, and hazardous alcohol use^2^. The impacts of behavioural risk factors may manifest as elevated blood pressure, elevated blood glucose, elevated blood lipids, and excess weight and obesity in individuals. These “intermediate risk factors” can be tested in primary care facilities and suggest a higher risk of heart attack, stroke, heart failure, and other consequences. These modifiable risk factors act on non-modifiable risk factors such as age, ethnicity, or genetic factors, to increase the risk of CVD.

Those born preterm possess a non-modifiable risk factor for cardiovascular disease (CVD) that is incurred at birth. Preterm birth is the birth of an infant at less than 37 completed weeks of gestation^5^. Preterm birth *per se*, that is, shortened gestation in and of itself, has been linked with an elevated CVD risk profile for over 20 years^6–10^, and is considered an independent risk factor for CVD^11,12^. While the risk is greatest for those born at the limits of viability (~22 weeks’ gestation), even those born moderate to late preterm exhibit an elevated risk profile^13^. However, preterm birth remains a poorly recognised risk factor of CVD, even among those born preterm^7,9,14^.

Due to the combination of animal models and human clinical and epidemiological studies we now have a comprehensive understanding of the gross structural and functional alterations in the preterm cardiovascular system. When taken together, these offer a means for lifelong monitoring of this known non-modifiable cardiovascular disease risk factor that is preterm birth. The purpose of this review is: 1) to explore the known structural and functional factors contributing to cardiovascular dysfunction and CVD across the early lifespan, and 2) to propose that persistent structural alterations are the dominant cause of preterm cardiovascular dysfunction and disease risk throughout life.

## Preterm trajectory of disease

Preterm birth is a complex and multifactorial phenomenon that can result from a combination of biological, environmental, and socioeconomic factors. In terms of biological factors, preterm birth can occur due to maternal medical conditions such as pre-eclampsia, infection, or cervical incompetence^5^. Additionally, genetic, and epigenetic factors can also play a role in preterm birth^15–17^. Furthermore, environmental factors such as socio-economic factors, poor nutrition, stress, exposure to pollutants, and substance abuse can also increase this risk of preterm birth^5^.

Preventive strategies for preterm birth include reducing risk factors such as maternal smoking and substance abuse, improving access to prenatal care, and promoting healthy behaviours such as good nutrition and stress management. However, preterm birth can have significant consequences for the infant, including an increased risk of neonatal morbidity and mortality, as well as long-term health consequences such as respiratory problems, cognitive and developmental delays, and an increased risk of chronic diseases in later life.

In recent years, prenatal impacts have been recognised as determinants of health and illness in later life, namely hypertension, ischaemic heart disease and heart failure^18–20^. Several epidemiological studies have demonstrated that prenatal and early childhood events may affect body composition and metabolism, thereby increasing the prevalence of several adult illnesses, including hypertension^21,22^, Type 2 diabetes^23,24^, and CVD^13,25^. Barker hypothesised that lower prenatal and postnatal growth may be associated with a higher risk of CVD in adulthood^26^. In addition, infants exposed to the Dutch Hunger Winter in early pregnancy during World War II were shown to have a higher incidence of obesity and cardiovascular disease later in life^27^. Following this, other studies established a definite association between preterm birth and CVD risk markers such as raised systolic and diastolic blood pressures^21,28^, impaired glucose tolerance and increased insulin resistance^23,29–31^, hypertriglyceridemia and low high-density lipoprotein levels in the blood^32,33^. Eriksson et al.^34^ looked at 4630 men born at Helsinki Hospital and found that men with a low ponderal index (the ratio of body weight to height) and slow weight gain in the first year of life had a greater risk of developing coronary heart disease later in life. Researchers found that premature babies were observed to experience ‘catch-up’ growth and had their body mass index go up between the ages of 1 and 12 were more likely to develop CVD^34^. However, this effect was only seen in children who had a ponderal index of 26 at birth^34^. Others have shown that low birth weight is a predictor of heart disease. However, low birthweight is an imprecise measure of growth in the womb and is not always caused by being born too early. Further research on preterm neonates has found that gestational age is a factor in the development of CVD^13,19,35,36^. In one study, men born preterm were shown to have greater quantities of total cholesterol, LDL-C, and apolipoprotein B than females^33^. Even after birthweight correction, these sexually dimorphic disparities in prematurely-born adolescents persisted^33^. When compared to full-term-born individuals, preterm birth was associated with higher LDL-C levels and elevated systolic and diastolic blood pressure^33^. Twin studies with dizygotic and monozygotic groups discovered that genetic and intrauterine environmental influences played a role in the development of CVD later in life^17,37^. Even though preterm birth is associated with a higher risk of developing CVD, the underlying processes or mechanisms that explain these correlations are not entirely known.

What is apparent is that this correlation with an elevated CVD risk profile is set from birth^38–41^. The capacity to adapt to the extrauterine environment determines survival in the immediate neonatal period, which has also been shown to have sexually dimorphic effects^42,43^. Male infants are born preterm at greater rates, exhibit more clinical complications during the neonatal period and are more likely to be readmitted following discharge than their female counterparts^43,44^. The cause of this discrepancy while unknown is likely multifactorial, with hormonal, genetic, and inflammatory factors playing key roles^44^. While more stable in the neonatal period, females born preterm exhibit an elevated risk profile throughout life with decompensation observable in adolescence^33^. While changes in neonatal care have significantly improved preterm survival, few improvements have eased the neonatal transition as much as the implementation of antenatal corticosteroids^45^. Indeed, while many infants now survive into adulthood without major comorbidities, all those born preterm carry a CVD risk inversely proportional to their gestational age^13,20,46^.

In addition to both maternal^5^ and neonatal factors^28,34^, antenatal corticosteroid (glucorticoid) treatments are increasingly associated with long-term disease outcomes^47,48^. Antenatal glucocorticoids have been routinely used since their introduction in the 1970’s to induce rapid lung maturation prior to birth^45,47^. While this treatment has become a mainstay treatment for prematurity – particularly at gestations <34 weeks (See Roberts et al.^49^) - the immediate effects on systemic growth may contribute to long-term cardiac, renal and insulin sensitivity^47,50,51^. Given the heterogeneity of the population, the long-term effects of antenatal corticosteroids remain conflicting, with late-preterm- and postnatal-administration appearing to add more controversy to this topic^52–54^.

### Infancy

At birth, foetal proliferation and development of the heart and arteries abruptly slows, interrupting the normal process of cardiomyocyte differentiation in preparation for postnatal life^55^. Animal studies have demonstrated smaller hearts with reduced number of binucleated myocytes following preterm birth^56^ (Table 1). Foetal hyperplastic cardiomyocyte growth of cardiac tissue is ceased by the transition to neonatal life, potentially limiting the lifelong myocyte size and number^38,48^. This phenomena impacts both the left and right ventricle, contributing to altered geometry of the heart, as well as affecting the heart’s contractile function and overall performance^20,57^. Additionally, mechanistic studies of the preterm ovine heart have demonstrated diffuse collagen deposition seven times greater than in term hearts^38,58^, and studies in mice have shown that the presence of short, disorganised myofibrils that fail to align in the myocardium in preterm models^59^. These structural changes incurred because of preterm birth persist beyond infancy and ultimately determine a greater risk of CVD during later life^20,60^.

**Table 1:** Systematic review of preterm cardiovascular changes in comparison to term cohorts from infancy through to adulthood

	Physiological observation	Physiological effect	Species	GA range*	Comment
*Infancy (neonatal)*					
Heart	Overall mass	↓^56,146^	H^146^, P^56^	34 weeks^146^, ~30 weeks E.^56^	
	LV mass	↔/↓^150^, ↑^38,56^	S^38^, P^56^, H^150^	~30 weeks E.^56^, 34 weeks^150^, ~35 weeks E.^38^	Isolated heart^38,56^, ↑ postnatal growth compared to term^150^ which appears inversely correlated to GA^151^
	RV mass	↑^38,56^	S^38^, P^56^	~30 weeks E.^56^, ~35 wees E.^38^	Isolated heart
	Binucleation	↓^56^	P	~30 weeks E.	
	Cardiac output	↓^70,71^	P	<30 weeks E.	Isolated heart (50% ↓)^70,71^, ↑ cardiac afterload and workload at 7 weeks^106^
	Contractility	↓^71^	P	<30 weeks E.	
	Adrenoceptor profile	∆^63^	P	23-28 weeks E.	↓ ß_1_/α_1A,_ ↑α_2A_^63^
Arteries	Aortic growth	↓^39^	H	28 weeks	Growth arrest at birth
	Arterial stiffness	↑^74^	H	<31 weeks	VLBW infants
Microvasculature	Blood flow	↑^42,62,78–81^	GP^79^, H^42,62,78,80,81^	27 weeks^81^, 29 weeks E.^79^, 24–36 wks^42,62,80^, 30-34 wks^78^	PU impaired/unstable when cold^85,152^
	Vessel density	↔^153,154^, ↓^76,77^	H	24-33 wks^154^, 32 wks^153^, 34 wks^76,77^	↓ due to PDA^76^, or functional perfusion^77^
	Pulmonary angiogenesis	↑^96^	H	23–29 weeks	Post-mortem analysis of short- vs long-term ventilation
Cardiovascular control	HRV	↓^64–66^	H^64–66^	25–37 weeks^64,65^, <28 weeks^66^	At TEA^64,65^, from birth through TEA^66^
	Microvascular (SNS)	↓^155^	H	24–36 weeks	In males, but not females
	Circ. Catecholamines	↔^84^, ↓^67,68^, ↑^69^	P^84^, S^68,69^, H^67^	27 weeks E.^84^, (130-131d GA)^68,69^, <32 weeks^67^	Responses to infused catechol.^68^
	BP	↓^71^	P	27 weeks E.	
Stress reactivity	Inotropes	↓^71^	P	27 weeks E	Dopamine/Dobutamine
	4% CO_2_ (HR, BP)	↑/↓^82,83^	H	27–34 weeks	Depending on severity of BPD
	Orthostatic challenge	↑^83^	H	29–33 weeks	
	Hypoxia	↓^84^	P	27 weeks E.	Despite similar catecholamine levels
	Temperature	↓^85^	H	23–28 wks	↓ vasomotor response to ↓ T_C_
*Childhood (5–10* *y/o)*					
Heart	LV mass	↓^94^	H	22–26 weeks	6 y/o
	RV mass	∆^95^	H	22–26 weeks	6 y/o
	RV thickness	↑^95^	H	22–26 weeks	6 y/o
	Contractility	↓^94,95^	H	22–26 weeks^94,95^	↑ wall stiffness^94,95^, LV and RV function altered
Arteries	Aorta diameter	↓^73^	H	22–26 weeks	
	Aortic stiffness	↑^93,108^	H	~27 weeks^93^, <32 weeks^108^	
	Coronary artery diameter	↓^73,89^	H	22–26 weeks^73^	↓ diameter at 6-and 9 y/o^73,89^
	Carotid thickness	↔^98^, ↓^73^	H	22–26 weeks^73^, 29 wks^98^	6 and 9 y/o
	Carotid stiffness	↓^73^	H	22–26 weeks^73^	6 y/o^73^
	Brachial artery diameter	↔/↓^145^	H	25–32 weeks	Marginal difference in diameter at 25-32 weeks^145^
	Brachial artery stiffness	↔^104,145,156^	H	25–32 weeks^145^, 29-32 weeks^104,156^	SGA, but not preterm birth, ↑ stiffness^104^
Microvasculature	Microvascular density	↓^90^	H	25–30 weeks	
	Retinal angiogenesis	↑^157^	H		
	Pulmonary vascular resistance	↑^95^	H	22–26 weeks	
Cardiovascular control	HRV	↔^64^	H	25–37 weeks	Investigated at birth, TEA, 2-3 y/o and 6-7 y/o
	BP	↑^90,93,145^	H	25–28 weeks^93^, 25-30 weeks^90,145^	7-12 y/o^90,93,145^
	Circ. Catechol.	↑^88^	H	27 weeks	9 y/o
Stress reactivity	Exercise capacity	↔^158^, ↓^91,92^	H	25 weeks^92^, 27-28 weeks^91,158^	Measured as power, V_O2_ (ml/kg), HR_max_, RQ^158^, primarily assessed resp. measures^91^
	Reactivity to ACH	↑^93^	H	~27 weeks	↑ LDF perfusion both initially and overall
*Adolescence (12–18* *y/o)*					
Heart	LV mass	↓^55^	H	28 weeks	
	LV size/volume	↓^55,119^	H	≤28 weeks^55,119^	
	LV function	↔^119^	H	<28 weeks	
	RV mass	↓^55^	H	28 weeks	
	RV size/volume	↓^55^	H	28 weeks	
Arteries	Aorta size	↓^119–121^	H	<28 weeks^119^, 29 weeks^121^	Adolescent girls^121^
	Arterial stiffness	↔^119^, ↑^109,159^	H	24–26 weeks^119^, 30 weeks^159^, 34 wks^109^	PWV btw carotid & brachial a.^59^, ↑ augmentation index for both SGA and AGA preterms^159^
	Intima-media thickness	↑^122^	H	25–28 weeks	Carotid intima-media in 12 y/o
Microvasculature	Vessel density	↑^122^	H	25–28 weeks	
	Endothelial function	↔^121^	H	29 weeks	
Cardiovascular control	BP	↔^159^,↑^33,109,121–124^	H	25–28 weeks^122,123^, 30 weeks^159^, 32–36 weeks^33^	↑ only in girls, but boys had ↑ atherogenic lipid profile^33^ SGA, but not AGA prematurity, ↑ SBP^159^
	Vascular resistance	↑^121^	H	29 weeks	
*Stress reactivity*	PORH	∆^122^			
	FMD	↔^10^	H	31 weeks	
	Exercise capacity	↓^124,160^	H	23–29 weeks^160^; 24-31 wks^124^	Aerobic capacity and strength^160^, reduced V_O2 MAX_ at 18 y/o^124^
*Young to Mid Adulthood (20–40* *y)*					
Heart	LV mass	↑^112,134^	H	30 weeks^112^, <36 weeks^134^	MRI-based study
	RV size/volume	↓^57^	H	30 weeks^57^	
	RV mass	↑^57,112^	H	30 weeks^57,112^	RV mass inversely related to GA^57^
	Myocardial fibrosis	↑^58,134^	H^134^, S^58^	<36 weeks^134^	
	Stroke volume	↓^112,134,149^	H	<30 weeks^112,149^	MRI-based study
	RVEJ	↓^57,112,161^	H	30 weeks^57,112,161^	
	Cardiac Output (RV)	↓^57^	H	30 weeks	SV significantly ↓, and HR ↑^57^
Arteries	Aortic diameter	↓^120,133,162^	H	27 weeks^120,162^, 30 weeks^133^	
	Aortic stiffness	↔^162,163^, ↑^110,111,133,164^	H	27 weeks^162^, ~30 weeks^110,133^, 32 weeks^93,111^, 34 wks^164^	High dependency on maternal pathology^110^, Inversely correlated with GA^91^, assessed via PWV^162,163^ 3 different assessment methods^133^
	Brachial/Carotid diameter	↔^133^	H	30 weeks	MRI-based cross-sectional area
	Carotid thickness	↔^162^, ↑^165,166^	H	27 weeks^162^, 29 weeks^166^, 33 weeks^165^	
	Brachial/Carotid stiffness	↑^162^	H	27 weeks^162^	
Microvasculature	Endothelial function	↓^165^		<37 weeks	
	Vascular density	↓^167,168^	H	30 weeks	Rarefaction expressed as ↓ branching points^167^ or expression of sENG/sFlt-1^168^
	Vascular resistance	↑^97^	H	28 weeks	
Cardiovascular control	HRV	↓^169–171^	H^169,170^, S^171^	28 weeks^169,170^, 36 weeks E.^171^	Reduced PNA^171^
	ECG	∆ Atrial activation^172^, ↑ QT^173^	H	28 weeks^172,173^	
	BP	↑^6–8,97,110,111,133,168,174–176^	H	28 weeks^97^, 30 weeks^110,133,168^, 32 weeks, 32-35 wks^175,176^	Sm. Physiological diff^6^; large ↑ in women^8^, HTN 2-3x more common^176^, ↑ pulmonary BP^97^
Stress reactivity	Exercise capacity	↓^97,129–131,177^	H	<30 wks^131^, <32 weeks^130,178^ ~ 29-36 weeks^129^	Multiparameter impairment^97,129,177^, Significantly ↑ BP during exercise^131^, SV ↓^130^
	Hypoxic pulmonary Vasoconstriction	↔^97^	H	28 weeks	
	Hypoxic response	↓^179^	H	29 weeks	Blunted microvascular response and ↑ oxidative stress
	Baroreflex	↑^180^	S	35 weeks E.	Late preterm sheep exhibited significantly ↑ BP response and ↓ HR response.
	FMD	↓^165^	H	<37 weeks^165^	Brachial a.^165^

Arrows (↓, ↑, ↔) indicate the direction of change relative to term controls. Species indicated by ‘H’ (human), ‘GP’ (guinea pig), ‘P’ (pig), ‘S’ (sheep). * The gestational age range of other species expressed as equivalent human weeks (denoted by ‘E.’). For methodology underlying systematic search of publications related to Table 1, refer to the Supplementary File.

The functional complications associated with premature transition pose a more immediate clinical significance during the neonatal period^61,62^. Studies of piglets have demonstrated altered adrenoceptor profile in the neonatal period^63^, which when combined with excess sympathetic tone^64–66^ and altered circulating catecholamines^67–69^, contributes to impaired cardiac output and cardiovascular instability^70,71^. Instability which is further exacerbated by alterations in both pulmonary and systemic vasculature. Persistent pulmonary hypertension is three times more common among preterms, impairing right ventricle ejection fraction^57,72^. Growth arrest and increased stiffness of the aorta increases afterload on the heart, further impairing cardiac function^39,73,74^ (Table 1). Patent ductus arteriosus (PDA) in many preterm neonates (perhaps as many as 50%^75^) also impedes attempts to improve cardiovascular stability in the neonatal period^76^. Furthermore, microvascular networks of preterm infants are rarefied and disorganised^76,77^, and are typically maximally dilated at rest^42,62,78–81^. These complications pose significant problems for the clinician as inotropes can prove fickle in rectifying circulatory failure (40% fail to respond to dopamine or dobutamine^71^).

**Fig. 1:**
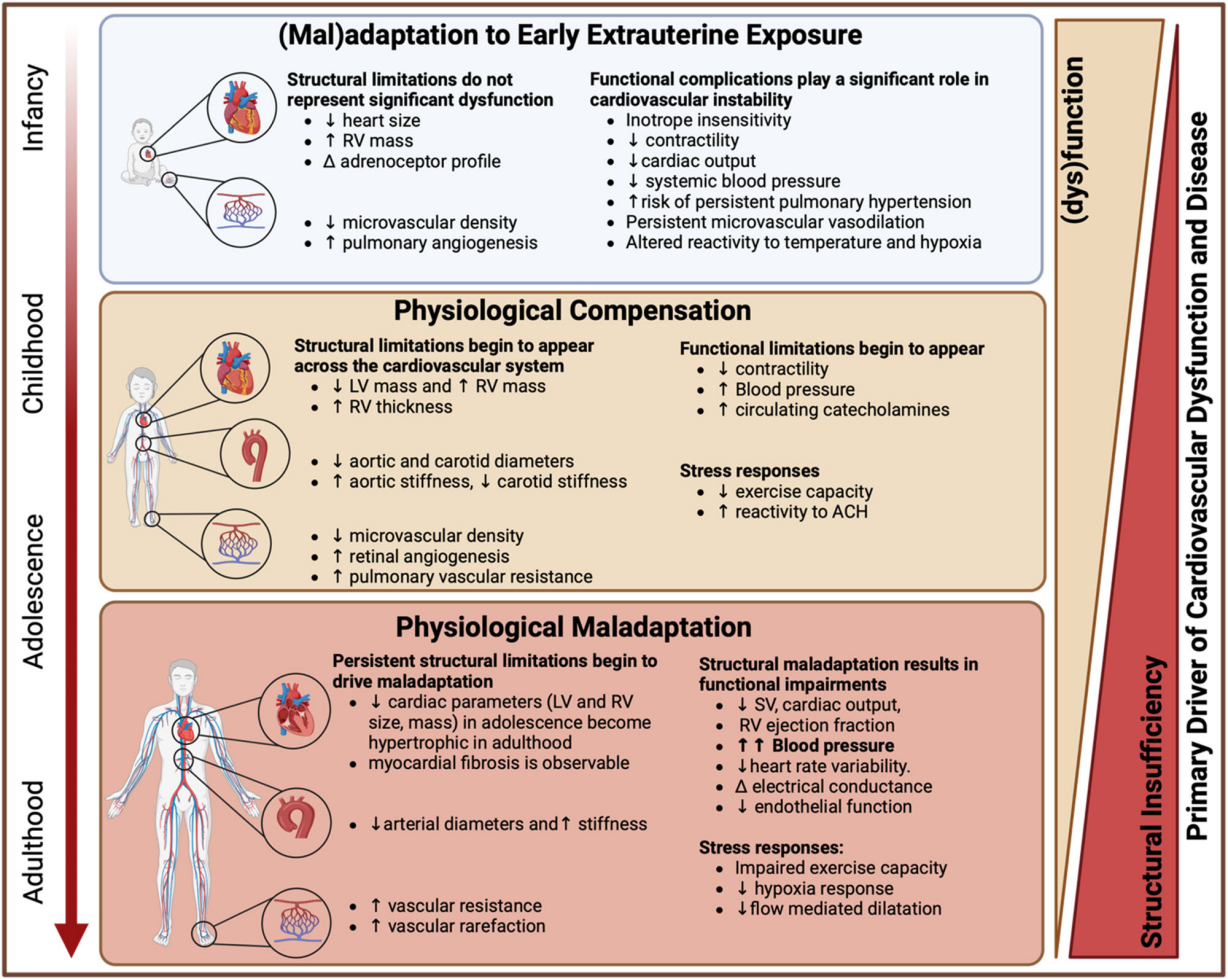
Relative contribution of structural and functional complications in preterm cardiovascular disease risk across the lifespan. Structural impairments (e.g., altered cardiac geometry^38,146^, ↓ microvascular density^76,77^) incurred with preterm birth ( < 37 weeks’ gestation) outside of clinically significant ones (e.g., patent ductus arteriosus) contribute relatively little to cardiovascular dysfunction, whereas functional complications (inotrope insensitivity^71^, ↑ microvascular perfusion^62^) are a significant cause of dysfunction (e.g., pulmonary hypertension^96,147,148^, ↓ cardiac output^70,71^). Surviving graduates of neonatal intensive care exhibit little cardiovascular dysfunction following discharge and throughout infancy. By childhood, structural limitations from prematurity become apparent (↓ LV mass^94^, ↑ RV mass^95^), resulting in some (dys)functional changes (↓ contractility^94,95^, ↑ BP^90,93,145^). Persistent structural limitations (altered cardiac geometry^55,57,119^, ↓ arterial diameter, ↓ microvascular density) contribute to cardiovascular remodelling (concentric hypertrophy^57,112,134^) and dysfunction in early adulthood (cardiac fibrosis^58,134^, arterial stiffness^111,149^, ↑ BP^28^). In combination with ‘traditional’ cardiovascular disease risk factors, these preterm-specific risk factors fuel the onset of disease.

Reactivity tests including exposure to 4% CO_2_, hypoxia, and thermal or orthostatic stress have elicited responses that are contradictory, but importantly, consistently altered from that of term-born infants^82–85^ (Table 1). These altered responses to stress may be a symptom of the heterogeneity of the preterm condition at different gestational ages and under different levels of clinical severity. However, they may also provide critical insight into cardiovascular stability in the neonatal period. As demonstrated by Stark et al.^62^, microvascular perfusion in the immediate postnatal period correlates with both cardiovascular stability and mortality within 72 h of birth. Preterm infants with greater vascular flexibility, and thereby improved stability, tend to have reduced clinical severity and better outcomes. As such, while many structural alterations are present within the neonatal period (e.g., PDA, reduced heart and artery size), the functional responses to neonatal life, and therefore the functional complications, appear to be of greater significance to neonatal morbidity and mortality (Fig. 1).

The acute transitional complications subside across the neonatal period as cardiovascular control matures^65^. Heart rate variability (HRV) studies have demonstrated maturation of cardiac control over the neonatal period such that by 2–3 years HRV is largely comparable between term and preterm infants^64–66^. Similarly, many of the functional issues such as aberrant dilation and cardiovascular instability resolve to a point where neonatal intensive care (e.g., inotropes, thermal support) is no longer required. Much of the clinical cardiovascular monitoring is frequently ceased following this neonatal period, with post-neonatal intensive care follow up focussing on critical neurodevelopmental milestones^86,87^.

### Effects of preterm birth on cardiovascular health throughout life

By childhood, the shortened gestation becomes apparent in the structure of the cardiovascular system. Whereas cardiovascular control appears comparable between term and preterm infants^64^, increased circulating catecholamines^88^, alongside narrowed arteries^73,89^ and reduced microvascular density^90^, results in elevated blood pressure (BP)^88,90^ and altered stress responses in children born preterm^91–93^. These structural and functional cardiovascular alterations seldom reach clinical significance, particularly following moderate-to-late preterm birth, but may be early markers of future disease present in childhood^36,94,95^.

Both left and right heart geometry remain altered in childhood, impacting cardiac contractility^36,94,95^. Using echocardiography with extremely preterm-born children, Mohlkert et al.^94^ demonstrated significantly smaller left ventricles and impaired ventricular function, which is associated with a 4-fold higher risk of heart failure in children and adolescents born between 28 and 31 weeks’ gestation and 17-fold increase with gestations below 28 weeks^36^. Investigating the right heart, Mohlkert et al.^95^ also demonstrated increased right ventricle thickness and altered geometry alongside increased pulmonary vascular resistance. While unable to parse out differences between functional versus structural causation, the alterations in the preterm right ventricle are likely due to a combination of reduced or immature pulmonary vessels, and the resultant increase in pulmonary pressure^96^. A similar mechanism is likely at play in the systemic circulation. Certainly, a history of pulmonary neonatal diseases is associated with an elevated risk of pulmonary hypertension by adulthood^72,97^. In the systemic circulation, preterm-born children show reduced aortic, coronary and carotid artery diameters^73,89^, though this is significantly affected by the length of gestation. At later gestations carotid artery size appears comparable to term-born children^98^, indicating a threshold effect to arterial compromise in childhood. Combining the work of Szpinda^99–101^, Zhong et al.^102^, and Schubert et al.^39^, the aorta grows linearly *in utero* with elasticity increasing significantly from 31 weeks’ gestation (remaining similar between 20 and 31 weeks’^102^), but aortic growth abruptly slows at birth. Impaired growth of carotid and coronary arteries has also been observed in extremely preterm-born children^73,89,98^, suggesting that growth cessation is common among major vessels.

While elastin accumulation is maximal in the perinatal period^103^, its synthesis in the aorta is significantly impacted by intrauterine growth restriction^104,105^ and presumably also by prematurity^74^. Furthermore, as collagen and elastin content remains almost constant from infancy up to 3 years^106,107^, failure to synthesise adequate amounts of elastin due to premature birth may permanently impact arterial compliance^103,106,108^. Indeed, Odri Komazec et al.^108^ identified decreased elasticity and increased stiffness in aorta of preterm-born children (<32 weeks’ GA), and these characteristics have been similarly observed in adolescence and adulthood following gestations of 30-34 weeks’^109–111^ (discussed further below). The altered compliance in the major arteries of preterm children is only exacerbated by reduced arterial diameters and microvascular rarefaction^90^. This is likely the cause of elevated blood pressure observed by Bonamy et al.^90^. The elevations in BP, while minor in childhood ( ~ 4 mmHg), further drive cardiac maladaptation and the propensity for disease formation.

The persistence of altered cardiovascular structure presents clinically and epidemiologically in adulthood. Studies by Lewandowski and colleagues^57,112^ show that the morphometric changes observed in preterm infants and children persist into young adulthood, with magnetic resonance imaging revealing significant differences in both left and right ventricular structure. Functionally, in two meta-analyses of preterm-born young adults, preterm birth was associated with 4.2 mmHg^113^ and 3.4 mmHg^114^ elevations in systolic BP, respectively, with both analyses noting stronger effects in women. Additionally, a recent large-scale study by Crump^13^, identified an adjusted hazard ratio of 1.28 and 2.45 for new-onset hypertension in preterm- and extremely preterm-born adults (18-29 y/o), respectively. Similarly, Risnes et al.^115^ observed a 1.4-fold and 1.2-fold increase in mortality in early and late preterm born individuals between 15 and 50 years. Supporting this, Crump et al.^28^ observed a significant relationship between preterm birth and prescription of antihypertensive medications in young adults (25–37 y/o). Preterm birth has been further linked with heart failure^36,116^, ischaemic heart disease^117,118^, and pulmonary vascular disease^97^, though this risk is strongly—and inversely—related to gestation^6,13^. In a register-based cohort study, Carr et al.^116^ observed a 17-fold increased risk of heart failure in those born extremely preterm (<28 wks’ GA), with this reducing to 3.6-fold in those born very preterm (28-32 wks’ GA). In terms of ischaemic heart disease, Crump’s register-based cohort study observed a 53% increased relative risk of developing ischaemic heart disease in preterm born individuals aged 30-43 years^117^. Such findings in those born preterm are perhaps unsurprising given the continuity of cardiovascular dysfunction from infancy, adolescence, and adulthood.

A clear trajectory of decompensation can be observed through adolescence and adulthood, precipitated by persistent structural alterations in the heart and vasculature of preterm-born children. The structural limitations, such as altered cardiac geometry, narrowed and rarefied vasculature become more pronounced by adolescence (Table 1). The hearts and arteries of preterm-born adolescents are smaller (LV^55,119^; RV^55^; aorta^119–121^), resulting in greater vascular resistance^121^ and elevated BP^33,109,121–124^. Many studies, though not all^98^, have also observed greater arterial stiffness and intima-media thickness; however, the causal mechanisms remain unknown. While data is sparse in adolescents, the elevated BP and vascular resistance do not appear to impair cardiac output (LV function^55,119^) or vascular function^10,122^ and no signs of concentric hypertrophy can be observed at this age^55^. However, by adulthood, those born preterm exhibit hypertrophic and functionally impaired hearts, narrowed and stiffened arteries, vascular dysfunction and rarefaction (Table 1). While much of the evidence is observed at gestations below 29 weeks, structural and functional alterations consistent with the overarching pathology are observable at later gestations (~34 weeks). Indeed, the conventional risk factors for CVD in young adults born preterm are often present across the spectrum of prematurity^13,108^. This unique aetiology of preterm-related CVD has driven calls for clinical recognition^14,18^, and potentially a new cardiomyopathy^36^.

## Stress reactivity

Stress tests are frequently employed to expose underlying cardiovascular dysfunction that is obscured at rest. Indeed, cardiopulmonary exercise testing is commonly used in the diagnosis of CVD. In populations with known risks of CVD, an impaired capacity to respond to – or recover from – the stressor may be indicative of early disease states. Stress testing may then provide useful prognostic insights into the preterm risk of CVD. Studies of those born preterm, from infancy through adulthood, have demonstrated persistent abnormal reactivity to a wide range of stressors, though there is ample room for expansion in these studies.

Autonomic and cardiovascular maturity has been examined in preterm infants using inotrope reactivity, hypercapnia, orthostasis and hypoxia (Table 1)^82–85^. Inotropes are routinely administered—with mixed efficacy—to improve cardiovascular compromise in hypotensive infants^125,126^. Mechanistic studies of preterm piglets have demonstrated reduced reactivity to both dopamine and dobutamine, with this possibly explained by immature cardiac and vascular adrenoceptor profiles (namely low abundance of cardiac ß_1_-adrenoceptors^63^ and vascular α-adrenoceptors^71^^,84^^,127,128^. As a result of immature neural control, preterm infants place greater reliance on circulating catecholamines; as demonstrated in the altered hypercapnic and orthostatic stress responses^82,83^, as well as hypoxic responses in preterm piglets^84^. Cohen et al. observed a 3- to 4-fold greater BP response compared to term-born counterparts with almost absent HR response when exposed to orthostatic stress^83^. Similarly, in response to hypoxia Eiby et al.^84^ observed a reduction in BP due to peripheral dilation with poor cardiac compensation. Together these studies demonstrate immature baro- and chemo-reflexes, particularly in the cardiac component of these reflexes. Notably, despite appearing stable at discharge from the hospital, these altered responses do not appear to resolve by term equivalent age^82^.

Childhood appears to be a deflection point in cardiovascular dysfunction when observed across the lifespan. Structural differences can be observed, but these appear to have a limited impact on function (Table 1). Exercise stress testing in children indicates a reduced exercise capacity, but due to a focus on respiratory function, limited inferences can be made to cardiovascular function^91,92^. One study in extremely preterm children exposed to acetylcholine challenge demonstrated elevated microvascular reactivity in children born appropriate for gestational age, though this only achieved significance compared to intrauterine growth-restricted preterm children and not term-born children^93^. Further studies in this age group would elucidate the impact of altered cardiovascular architecture and may prove beneficial for identifying early markers of disease.

By early adulthood, the structural limitations in the preterm cardiovascular system begin to produce pronounced dysfunction during stress testing. Using stress echocardiography, Huckstep et al.^129^ demonstrated progressive impairment in left ventricular ejection fraction and cardiac output during graded exercise, with this likely due to altered cardiac geometry exhibited at rest^112,129^. In a similar study, Macdonald et al.^130^ demonstrated impaired stroke volume augmentation and impaired right heart kinetics during exercise. This resulted in increased cardiac work for comparable stroke volumes and an increased reliance on heart rate response^130^. Importantly, these significant changes exhibited under stress were not present at rest^130^. Furthermore, examination of the vasculature during stress testing has shown increased stiffness in the form of increased pulse wave velocity, systolic blood pressure and pulse pressure in the brachial artery^131^. Increased vascular stiffness has been shown to increase afterload, and increase cardiac work for a given stress^132^, though the changes in preterm vasculature do not necessarily reach clinical significance^133^. This may explain the hypertrophic changes in both left and right ventricles^57,112,134^. Recovery, too, is impaired with preterm adolescents and adults exhibiting impaired heart rate recovery following graded exercise^135,136^. Heart rate recovery following exercise is primarily due to parasympathetic activation and sympathetic withdrawal^137^, and its impairment has been shown to be a predictor of cardiovascular disease^135,137^. Finally, in a cohort of preterm-born adults a 16 week exercise training intervention improved aerobic capacity and power, but not ambulatory systolic or diastolic blood pressure, a major risk factor for CVD^138^.

Collectively, the above studies demonstrate a trajectory of dysfunction as a result of persistently altered cardiovascular structure. The alterations in structure become progressively deleterious with age such that by early adulthood the altered cardiac structure exhibited at rest produces functional impairment under stress. Such studies demonstrate both the efficacy of stress testing in the preterm population as well as the value of stress testing as a prognostic test. While there are indications of system-wide dysfunction at rest, in the form of altered cardiac structure and vascular diameter and stiffness, the effects of these alterations appear dysfunctional under stress.

## Current and future directions

As discussed in the outset, calls have been made for preterm birth to be recognised as a non-modifiable risk factor for cardiovascular disease for over 20 years now^9,12,36,139^. As a non-modifiable risk factor, short of preventing preterm birth, the root cause cannot be treated. Furthermore, the pathophysiological mechanisms contributing to CVD in preterm-born adults remains undetermined^36,139^. It appears, however, that structural insuffiencies strongly contribute to CVD risk (Fig. 1, Table 1), with contributing factors across multiple systems^140^. For graduates of neonatal intensive care, many are discharged from neonatal follow-up programmes early on, which consist mainly of neurodevelopmental milestones^14^. However, given the weight of evidence supporting lifelong risk of chronic disease including – but not limited to – CVD, and the absence of treatments, a sustained cardiometabolic follow-up programme offers a cost-effective and practical solution^139,140^. In ‘traditional’ CVD populations, acknowledging non-modifiable risk factors (e.g., family history), educating patients, and advising lifestyle interventions (e.g., diet, exercise, smoking cessation) are proven treatment options – used alone or in combination with pharmacological interventions to treat CVD^141–144^. Indeed, a recent questionnaire by Girard-Bock et al.^14^, found that many of the preterm-born adults were not even aware of their heightened CVD risk. They concluded that it is essential that long-term consequences of preterm birth are effectively communicated to preterm-born populations^14^. They, among others, noted that preventative strategies would be an effective treatment in the preterm population^14,139,140^. Current guidelines for BP management call for non-pharmacological management in patients with systolic BP of 120-139 mmHg^140,144^. While pharmacological treatment has been demonstrated to be effective in patients with systolic BP between 130-139 mmHg, it has not been recommended for young adults^140,144^. Elevated BP can be detected in the preterm population from childhood^90,93,145^, with dysfunctional traits manifesting in early adulthood (Fig. 1, Table 1). Jones et al.^140^, recommended heightened monitoring, including at-home BP measurement and early counselling on lifestyle interventions, with pharmacotherapy an option in high-risk patients. Given the efficacy of preventative strategies in other populations, such an approach will certainly save more in the long-run than waiting for the disease to manifest.

## Conclusion

Events that alter the normal trajectory of early life development have profound implications for life-course health and wellbeing extending decades beyond the insult. Those born preterm are a heterogenous group in terms of sex, gestation, and neonatal morbidity. However, two things are clear: 1) preterm birth produces permanent structural changes to the heart and vasculature; and 2) preterm birth is associated with long-term risk of CVD.

Here, we have put forward the hypothesis, that the structural limitations incurred at birth produce adverse functional cardiovascular changes which, across the lifespan, drive maladaptive remodelling (e.g., concentric cardiac hypertrophy, arterial fibrosis) and CVD (Fig. 1). Such changes are pivotal stages in ‘traditional’ CVD aetiology. The key difference between the ‘traditional’ and preterm populations is that those born preterm require no further insult (e.g., poor diet, smoking, stress) to drive CVD, as the persistent structural changes drive hypertension, impaired cardiac output, and endothelial dysfunction.

## Supplementary information

Supplemental file-Table 1 methods
