# Supplementary material for: Impact of prematurity on lifelong cardiovascular health: structural and functional considerations
Source: NPJ Cardiovasc Health. 2024 Apr 5;1:2. doi: 10.1038/s44325-024-00002-0 (PMC12479354; doi:10.1038/s44325-024-00002-0)
Supplement: Supplementary file 1 — Supplemental file-Table 1 methods [file 44325_2024_2_MOESM1_ESM.pdf]

For the literature reviewed in Table 1

A systematic search was conducted in electronic databases (PubMed, Medline via Ovid, and Cochrane Library) using predefined keywords: “cardiovascular disease”, cardiovascular dysfunction”, “cardiovascular”, “vascular”, “artery”, “blood pressure”, “hypertension” and “infant, premature”, “premature birth”, “preterm”, “preterm-born”, “infant”, “childhood”, “adolescence”, “adulthood”. The search was conducted up to April 2023 (Searches not directly related to Table 1 continued until January 2024). Searches were limited to English language. No date range was included for publications. Hand searching of bibliographies was conducted to identify candidate papers not initially screened. Where a cardiovascular characteristic was missing at a particular age range (e.g., aortic diameter), a secondary search (e.g., “aorta”, “childhood”, “preterm”) was conducted in an effort to obtain continuity of physiological characteristics among age groups.

Searches were conducted between May 2022 and April 2023. Inclusion criteria were as follows: (1) comparison of preterm-born (<37 weeks or equivalent in relevant animal model) and term-born subjects, (2) reporting of cardiovascular structure and/or function (e.g., aortic diameter and/or elasticity), (3) subjects were free of significant congenital malformations or acute illness. An annotation under ‘comment’ is included where small for gestational age (SGA) or low to very low birth weight infants (LBW or VLBW) are included within studies, either as a mixed cohort, or where this definition is used as the primary inclusion criteria, with shortened gestation being a naturally occurring cofactor of low-birth weight in the absence of pathological fetal growth restriction. Of the 108 studies matching the inclusion criteria, a further 11 were excluded as they primarily addressed other systems (pulmonary function, pain pathways, hypothalamic-pituitary-adrenergic pathway), or did not provide adequate

comparison to term born controls, or did not define prematurity separately from intrauterine growth restriction. The final date range of included publications was between 1985 and 2023. Of interest, the earliest screened experimental study was by Moss et al., 1963 [1], though this was excluded due to changes in the defining characteristics of prematurity subsequent to their publication.

1 Moss, A. J., Duffie, E. R. J. & Emmanouilides, G. Blood pressure and vasomotor reflexes in the newborn infant. *Pediatrics* **32**, 175-179 (1963).
